# Fragmented QRS complex in athletes’ electrocardiogram: physiological adaptation or pathological sign? A scoping review

**DOI:** 10.1186/s43044-026-00730-x

**Published:** 2026-04-16

**Authors:** William Wiradinata, Faqrizal Ria Qhabibi

**Affiliations:** 1 Faculty of Medicine, University of Brawijaya, Malang, Indonesia; 2 Department of Cardiovascular Prevention and Rehabilitation, National Cardiovascular Center Harapan Kita, West Jakarta, Jakarta, Indonesia

**Keywords:** Athlete, Electrocardiogram, Fragmented QRS, Preparticipation screening

## Abstract

**Background:**

Fragmented QRS (fQRS) complex, characterized by notching/slurring in the QRS complex, has been linked with myocardial fibrosis and sudden cardiac death (SCD) in cardiomyopathies. While fQRS is a recognized arrhythmogenic marker in hypertrophic cardiomyopathy, it is not included in the International Criteria for ECG interpretation in athletes.

**Methods:**

This review aims to evaluate the prevalence and clinical significance of fQRS in athletes. This review was done according to the Preferred Reporting Items for Systematic Reviews and Meta-Analyses Extension for Scoping Reviews (PRISMA-ScR) guideline. A search was conducted across PubMed, ScienceDirect, and Google Scholar to identify relevant studies reporting fQRS in athletes across all age groups and sports discipline.

**Results:**

A total of 7 studies were included in the final review, revealing higher prevalence of fQRS in older age groups, with the highest prevalence occurring in lead V1. Athletes with fQRS also experienced no adverse events on follow-ups.

**Conclusions:**

Current data suggests that isolated fQRS, especially in lead V1, is likely benign. However, fQRS in ≥ 2 contiguous leads warrant further investigations. A longer follow-up duration is needed to ascertain the possibilities of adverse events occurring in athletes.

**Supplementary Information:**

The online version contains supplementary material available at 10.1186/s43044-026-00730-x.

## Background

Prevention of sudden cardiac death (SCD) in active populations, competitive or not, has always been an important point in sports cardiology. Preparticipation screening (PPS) guidelines specifically aimed at detecting cardiac abnormalities, such as the Seattle and International Criteria, helps in interpreting electrocardiograms (ECGs) of athletes, categorizing various ECG manifestations based on its relationship to physiological cardiac adaptation to exercise [1, 2]. Besides the suggestion made by the European Society of Cardiology (ESC) to employ ECG as an initial part of PPS, a study conducted by Corrado et al. strengthens the reasoning for the implementation of ECG as an important aspect in PPS, indicating that systematic ECG screening significantly decreased the SCD rate from 3.6 to 0.4% [3]. Over the past two decades, the standards for ECG interpretation in athletes have been enhanced and widely disseminated. Latest evidence indicates that QRS fragmentation in arrhythmogenic cardiomyopathies may hold clinical relevance concerning SCDs [4].

Fragmented QRS (fQRS) complex has been linked with myocardial fibrosis, which occurs in various cardiac abnormalities [5, 6]. One of the cardiac abnormalities linked with myocardial fibrosis is hypertrophic cardiomyopathy (HCM), one of the leading cause of SCDs in athletes [7, 8]. The sensitivity and specificity value of fQRS in detecting myocardial fibrosis in HCM patients was reported to be 84.6% and 90%, respectively [8]. fQRS is also linked with an increased risk of SCDs [9]. Myocardial fibrosis in HCM may develop as a secondary effect of cardiomyocyte hypertrophy and the early activation of profibrotic signalling pathways during the disease's progression [10].

Although fQRS has a significant correlation related to myocardial fibrosis in HCM, it is currently not mentioned in the International Criteria for ECG interpretation in athletes. Therefore, this review aims to reveal the clinical significance of fQRS in athletes, examining the prevalence, demographic data, the type of sports practiced, its associations with other cardiac abnormalities present in athletes, and the occurrence of adverse cardiac events.

## Methods

This scoping review followed the Preferred Reporting Items for Systematic Reviews and Meta-Analyses Extension for Scoping Reviews (PRISMA-ScR) framework [11].

### Search strategy

A search was conducted from March 2nd to March 14th, 2025 across three electronic databases: PubMed, ScienceDirect, and Google Scholar [12]. The keywords used in the search were “fragmented QRS” and “athlete”. Two reviewers independently screened the search results based on the title and abstract, followed by a full-text assessment for eligibility. Disagreements during screening were addressed through consensus-based discussions between the reviewers.

### Eligibility criteria

We included articles examining the presence of fragmented QRS in athletes of all sports disciplines and age groups with no current or previous cardiovascular diseases. The analysis was restricted to articles published in English to maintain accessibility and ensure inclusion of widely accessible research. Eligible study designs included randomized controlled trials, cohort studies, and case-based research such as case reports, case series, or case presentations.

### Data extraction and quality analysis

To minimize bias and enhance the credibility of the findings, two independent reviewers assessed the eligibility and methodological rigor of the selected studies. Both researchers also carried out systematic data extraction. Any disagreements during the evaluation process were addressed through consensus-based discussions to ensure consistency.

## Results

### Study selection

**Fig. 1:**
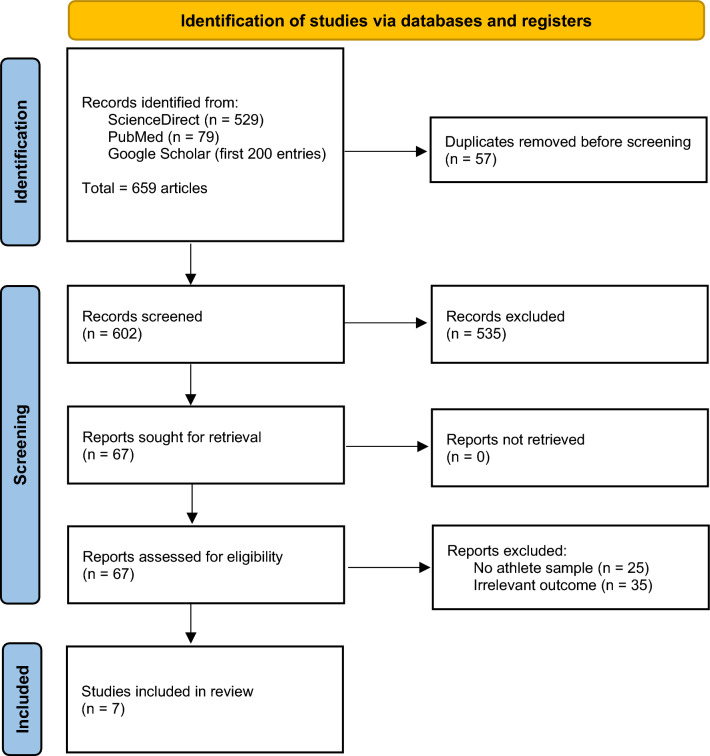
Flowchart describing the article selection process

The initial search yielded 659 articles, comprising 529 articles from ScienceDirect, 79 articles from PubMed, and the first 200 entries from Google Scholar. Following the removal of duplicate entries, 602 articles remained for evaluation. After reviewing titles and abstracts, 67 full-text articles were selected for further eligibility assessment. A total of 25 articles were excluded due to the wrong sample population, and 35 articles were excluded due to irrelevant outcomes. Ultimately, 7 articles [13–19] met the inclusion criteria and were incorporated into the final analysis. A detailed overview of the selection process is provided in Fig. 1.

### Characteristics of population and included studies

**Fig. 2:**
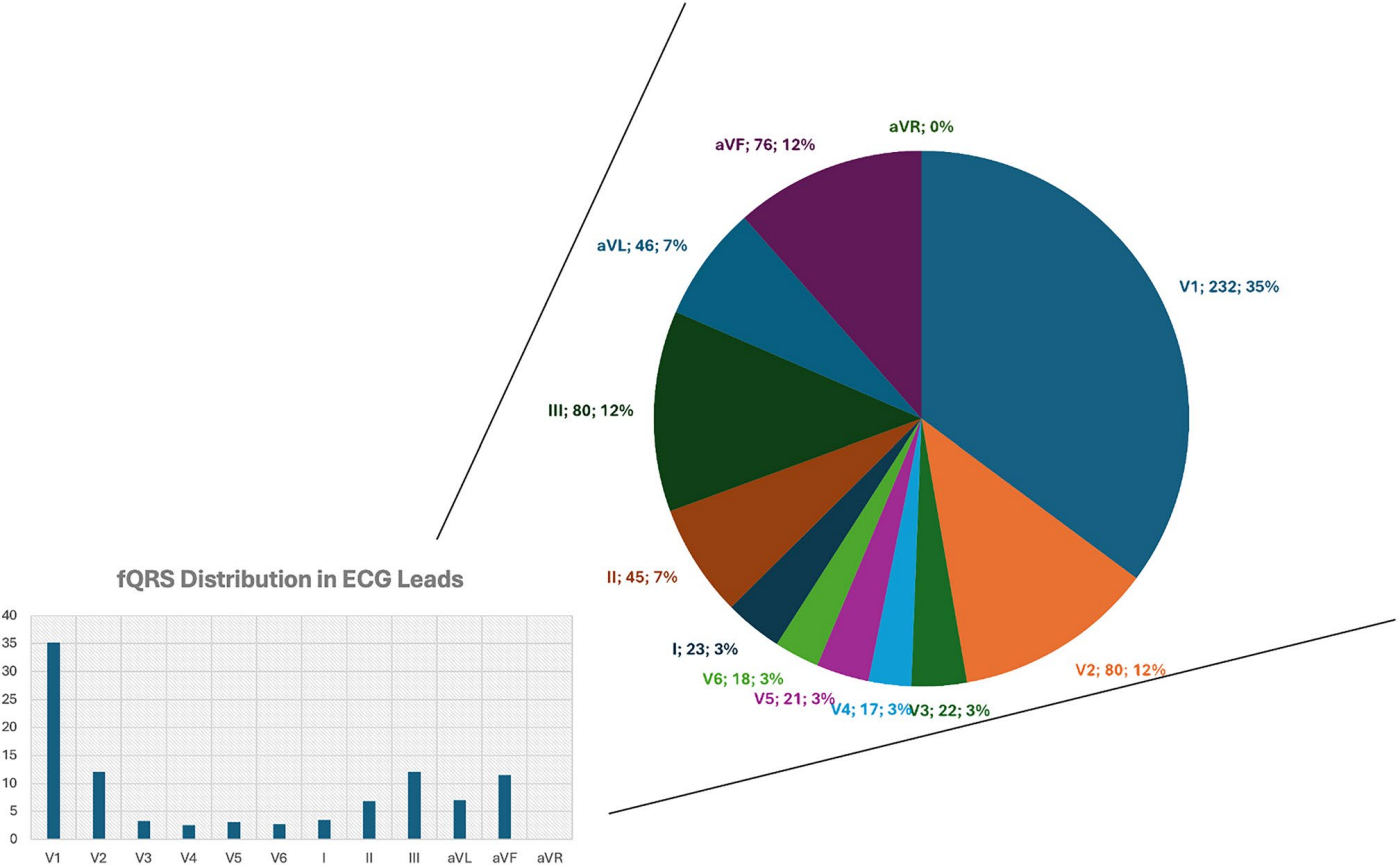
Distribution of fragmented QRS in ECG leads

Seven observational studies, involving a total of 4071 athletes, were included in the final analysis. The study participants included athletes from diverse age categories. The sports branches of athletes included in the study consists of various sporting disciplines, usually categorized according to ESC recommendation or Italian Cardiological Guidelines (COCIS). The detailed characteristics of samples within included studies could be seen at Supplementary Tables S1 and S2 (Fig. 2).

### Fragmented QRS complex in athletes

Fragmented QRS (fQRS) is characterized by an extra notch in the QRS complex without the presence of a bundle branch block. The electrophysiological mechanism responsible for QRS fragmentation is a complex and heterogeneous cardiac excitation resulting from structural or anatomical abnormalities, such as myocardial fibrosis [15, 20]. While fQRS presence lacks clinical significance for mortality in healthy individuals, it holds considerable value in athletes, as the potential for an electrocardiographic pattern linked to regular exercise must always be considered. Moreover, physical exertion, especially with vigorous intensity, presents an increased risk of SCDs in individuals with undetected heart disease, requiring a low threshold for comprehensive diagnostic assessment upon the identification of a concerning electrocardiographic pattern. Nevertheless, only a few research have examined the clinical implications of fQRS in athletes, as the predominant body of medical literature on this topic has concentrated on individuals with established diagnosis of cardiac disease.

The occurrence of fragmented QRS is primarily observed in lead V1, followed by lead III and aVF. The occurrence of fragmented QRS in other leads are comparatively rare. Based on lead categorization, fragmented QRS primarily occur in the right precordial and inferior leads. A detailed breakdown of the data is presented in Table 1. A study utilizing cardiac magnetic resonance (CMR) reveals that in 48% of athletes diagnosed with myocardial fibrosis, the late gadolinium enhancement (LGE) is confined to the septum or right ventricle (RV) insertion points, similar to those found in pulmonary arterial hypertension patients. This could be caused because exercise induces a more substantial relative elevation in pulmonary systolic pressure compared to aortic systolic pressure, leading to a 125% rise in wall stress for the RV compared to a 4% increase for the left ventricle (LV) [21, 22]. The thinner wall of the RV may promote the progression from elevated wall stress to cardiomyocyte injury more than in the LV, potentially explaining the higher prevalence of fQRS occurring in the septal and anterior leads especially in V1 among athletes.

**Table 1:**
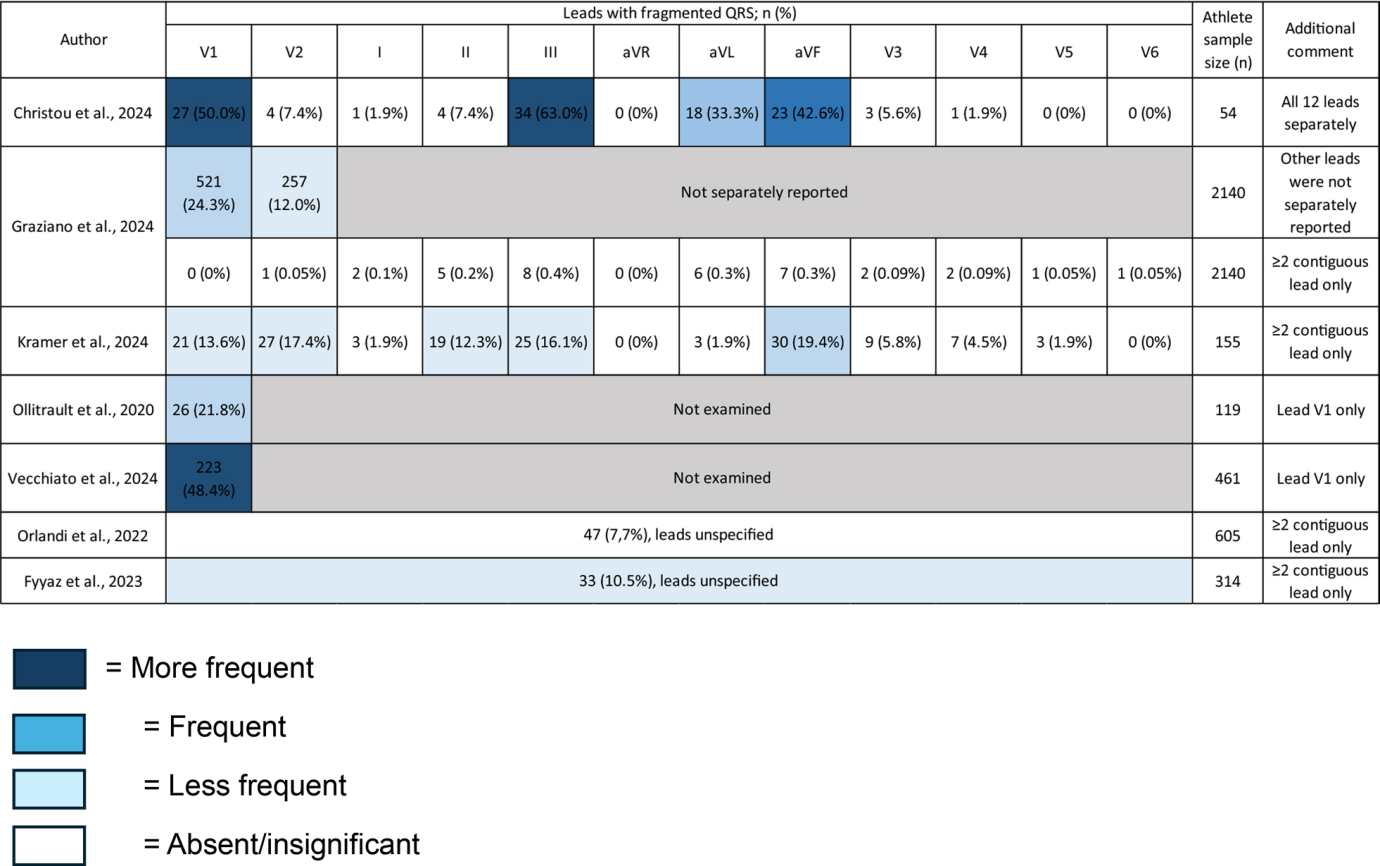
Frequency of fragmented QRS in athletes

A study by Graziano et al. involving 9 athletes with fQRS in over two contiguous leads, who subsequently underwent exercise testing and follow-up, revealed no abnormalities. However, echocardiography in 1 athlete with fQRS in more than two contiguous leads identified mitral valve prolapse with mild regurgitation [13]. A study by Orlandi et al. and Kramer et al. showed that the occurrence of fQRS in over two contiguous leads tends to occur in older age groups or masters athletes [14, 15]. This finding is compounded by the study done by Graziano et al. in young athletes (7–18 years) with the occurrence of fQRS in ≥ 2 contiguous leads only amounting to 0.5% [13]. The types of sports and training loads did not affect the occurrence of fQRS in athletes; however, fQRS was significantly associated with lower heart rate, left ventricular wall thickness, and cardiac mass index. fQRS are also more prevalent in males, although this finding could be caused by the predominantly male cohort, thus not reflecting real correlation to gender.

Regarding the occurrence of adverse cardiac events, follow-up was conducted by Graziano et al., Vecchiato et al., and Christou et al. with varying durations [13, 16, 17]. The results of the follow-up revealed no occurrence of adverse cardiac events or any SCDs occurring in athletes with fQRS. Other ECG abnormalities are also most likely unrelated to the occurrence of fQRS, as shown by Fyyaz et al. and Vecchiato et al., showing no significant difference between athletes with fQRS in ≥ 2 contiguous leads and fQRS only in lead V1 to athletes without fQRS regarding the occurrence of other ECG based abnormalities [16, 18].

## Discussion

### Exercise—myocardial fibrosis—SCDs network

Exercise-induced cardiac remodeling is characterized by biventricular dilatation accompanied by supranormal diastolic function, bi-atrial dilation, and improved arterial vasomotor function. These adaptive characteristics promote the preservation of elevated cardiac output during endurance exercise and may prevent age-related detrimental cardiac deconditioning [23]. However, prolonged rigorous exercise may exceed adaptive thresholds, provoking pathological remodeling such as myocardial fibrosis. Myocardial fibrosis is characterized by a substantial increase in the collagen volume of myocardial tissue. The process is intricate, encompassing all elements of cardiac tissue, and can be initiated by tissue damage due to myocardial ischemia (hypoxia), inflammation, and hypertensive stress [24–26]. The pathophysiological mechanisms of myocardial fibrosis in athletes varies substantially. The increase in cardiac troponin I and T in a study involving marathoners suggests that repeated exposure to endurance exercise may cause cardiac microdamage, which may contribute to the development of myocardial fibrosis following prolonged exercise training, particularly in veteran athletes [27].

Cardiomyopathies constitute the primary cause of mortality in athletes at 62%, with most cases associated with myocyte hypertrophy and fibrosis [28]. A study conducted by de Noronha et al. stated that 73 athletes, constituting 62% of those with SCDs, were found to have fatalities linked to a primary myocardial illness (cardiomyopathies). Furthermore, left ventricular hypertrophy was the predominant abnormality observed on macroscopic examination. Left ventricular hypertrophy is also correlated with histological findings of myocardial fibrosis [29]. This issue was highlighted in a recent case report of a marathon runner who passed away during the race due to left ventricular hypertrophy and extensive myocardial fibrosis [30]. This condition may indicate acquired cardiomyopathy associated with exercise-induced arrhythmias.

### Development of fragmented QRS complex in myocardial fibrosis

Fragmented QRS (fQRS), defined by revealed notching or slurring in the QRS complex without a bundle branch block on a 12-lead ECG, indicates conduction delay resulting from myocardial scar tissue, including myocardial fibrosis or infarction. Prior research indicated that fQRS may serve as a more sensitive indicator than pathological Q waves for identifying myocardial fibrosis, as evaluated by perfusion scintigraphy [31]. Numerous investigations have revealed a robust correlation between fQRS and myocardial fibrosis, evaluated using gadolinium-enhanced CMR [32, 33]. Cardiac fibrosis may extend cardiac conduction time, thereby altering the shape of the QRS complex and resulting in the emergence of fQRS.

A study by Konno et al. in 2015, who identified fQRS in patients with HCM, reveals that it primarily occurs in the inferior leads, followed by the anterior and lateral leads, whereas pathological Q waves were mainly found in the lateral leads [31]. Consequently, fQRS manifests more frequently than pathologic Q waves in all segments. Related to the presence of fQRS as a manifestation of myocardial fibrosis, fQRS demonstrated superior sensitivity compared to pathological Q waves in identifying cardiac fibrosis [31]. Nonetheless, there is a compromise in specificity; fQRS in inferior leads exhibited significantly lower specificity compared to pathologic Q wave. In this context, both extensive myocardial scarring and micro focal fibrosis have been histologically seen in cardiac remodeling. Microfoci of fibrosis are undetectable by LGE-CMR or pathological Q waves; yet, these diminutive fibrotic tissues may induce conduction delays, which likely accounts for the reduced specificity of fQRS compared to pathological Q waves in myocardial remodeling among athletes [31, 34].

The relationship between fQRS and myocardial fibrosis highlight significant concerns regarding the biological mechanism of left ventricular remodeling and arrhythmogenicity in HCM [35]. Elevated myocardial fibrosis leads to left ventricular systolic dysfunction and heart failure, while also potentially creating additional arrhythmogenic anatomical pathways. These findings align with previous studies that have shown correlations between fQRS and the incidence of arrhythmic events in patients with HCM [36]. Incorporating fQRS into conventional risk variables may enhance the prediction accuracy for sudden cardiac death in athletes.

Although fQRS is a sensitive marker of fibrosis in populations with underlying cardiac abnormalities, our data suggests that the occurrence of fQRS in athlete population lacks pathological specificity. The occurrence of fQRS in HCM patients observed by Konno et al. are more frequently encountered in the inferior leads, compared to the anterior and lateral leads, compared to our findings where fQRS predominantly occur in lead V1, which suggests the involvement of RVOT adaptation. The possibility of micro focal fibrosis in athletes, undetectable by LGE-CMR, could be one possible explanation regarding fQRS in athletes, but the absence of any adverse events during follow-up undermines its clinical significance.

### Clinical implication of fragmented QRS in athletes

Fragmented QRS complex, indicative of scar tissue, signifies substantial underlying cardiac pathology with an arrhythmogenic component. The presence of fQRS can be an arrhythmogenic marker in various clinical conditions such as ischemic cardiomyopathy, non-ischemic cardiomyopathy, Brugada syndrome, and hypertrophic obstructive cardiomyopathy (HOCM) [37]. In ischemic cardiomyopathies, the correlation between fQRS with perfusion and functional abnormalities is higher compared to the Q wave, and fQRS may indicate a previous silent myocardial infarction (MI). In terms of predicting remote MI, the sensitivity of the Q wave (36.3%) was less than half compared to fQRS (84.6%) [37].

Fragmented QRS is related with intraventricular dyssynchrony in non-ischemic cardiomyopathy patients exhibiting a narrow QRS interval, where this irregular ventricular activation may result in arrhythmias. Research indicates that the fQRS complex had excellent sensitivity (90.6%) and negative predictive value (85%) for detecting intra-ventricular dyssynchrony in patients with non-ischemic dilated cardiomyopathy (DCM) and a narrow QRS complex [38]. Moreover, premature ventricular complexes (PVCs) in the absence of underlying heart disease are linked with the occurrence of ventricular tachycardia. Research indicates that the occurrence of fQRS correlates with frequent PVCs in individuals devoid of apparent structural cardiac disease [39]. Consistent with that assertion, Sha et al. demonstrated that the prevalence of ventricular tachyarrhythmias and all-cause mortality in individuals exhibiting fQRS was markedly elevated compared to those lacking fQRS on their ECG (23.5% vs. 3.4%, *p* = 0.043) [40].

HOCM poses a significant SCD risk in young, asymptomatic individuals, including trained athletes. fQRS is significantly prevalent in patients with HOCM, and its presence correlates with a poorer prognosis, indicating a higher risk of arrhythmic events. Magnetocardiographic analysis of 11 patients (10 exhibiting fQRS) with HOCM, narrow QRS complexes, and implantable cardioverter-defibrillators (ICDs) demonstrated a significant prolongation of left ventricular conduction time in HOCM patients relative to controls [35].

While fQRS signifies risk of arrhythmia in populations with cardiomyopathy (HCM and DCM), its implications may differ in athletes. While Sha et al. found that fQRS predicts intraventricular dyssynchrony and mortality in the general population, our findings show that asymptomatic athletes experienced no occurrence of adverse events. Current evidence suggests that the occurrence of isolated fQRS, especially in lead V1, might be benign, while the occurrence of fQRS in ≥ 2 contiguous leads require further investigations, without forgetting other ECG related manifestations that occur.

## Limitations

One of the limitations in this review is the lack of data regarding CMR utilization in athletes with fQRS in order to confirm the presence of fibrosis and other possible cardiac abnormalities. The male dominated sample base also may skew the findings, possibly not reflecting true association between fQRS and sex. Another notable limitation is the diverse sample base including various sporting disciplines and different levels of competition within the sport, warranting caution when extrapolating the data to general athletic population. Current results found no adverse events occurring in athletes during the follow-up duration, but the possibility of adverse events occurring in a longer follow-up time frame remains.

## Conclusion

Fragmented QRS is a straightforward, cost-effective, and accessible ECG indicator that can be easily interpreted. Numerous prior investigations have investigated fQRS as a diagnostic indicator and its capacity to forecast arrhythmic episodes in both ischemic and non-ischemic conditions, where arrhythmia is a characteristic consequence. fQRS may be crucial in identifying athletes with concealed cardiac disorders, enabling urgent treatment to avert life-threatening arrhythmias that could result in SCD. Current evidence suggests that isolated fQRS could be benign, whereas fQRS in ≥ 2 contiguous leads require further investigations.

## Supplementary Information

Supplementary Material 1.

## Data Availability

No datasets were generated or analysed during the current study.

## Abbreviations

CMR: Cardiac magnetic resonance imaging
COCIS: Italian Cardiological Guidelines
DCM: Dilated cardiomyopathy
ECG: Electrocardiogram
ESC: European Society of Cardiology
fQRS: Fragmented QRS
HCM: Hypertrophic cardiomyopathy
HOCM: Hypertrophic obstructive cardiomyopathy
ICD: Implantable cardioverter-defibrillator
LGE: Late gadolinium enhancement
LV: Left ventricle
MI: Myocardial infarction
PPS: Preparticipation screening
PRISMA-ScR: Preferred Reporting Items for Systematic Reviews and Meta-Analyses Extension for Scoping Reviews
PVC: Premature ventricular complex
RV: Right ventricle
SCD: Sudden cardiac death
