# Supplementary material for: Fragmented QRS complex in athletes’ electrocardiogram: physiological adaptation or pathological sign? A scoping review
Source: Egypt Heart J. 2026 Apr 16;78:24. doi: 10.1186/s43044-026-00730-x (PMC13086978; doi:10.1186/s43044-026-00730-x)
Supplement: Supplementary file 1 — Supplementary Material 1. [file 43044_2026_730_MOESM1_ESM.docx]

Table S1. Characteristics of studies included

| Author | Year | Study Design | Sample Size (n) | Demographic | Sport Types | CMR Test | Training Load |
| --- | --- | --- | --- | --- | --- | --- | --- |
| Graziano et al. [13] | 2024 | Retrospective | 2140 | Young athletes aged 7-18 years old, 49% were male, and 98.5% were Caucasian; mean age: 12.5 ± 2.6 years | Based on ESC recommendation:  • mixed sports (61%)  • endurance sports (29.1%)  • skill sports (9.9%) | Only conducted in 1 patient with fQRS in ≥2 contiguous leads with no abnormalities found | • >6 hours per week (25.6%)  • 4-6 hours per week (35%)  • <4 hours per week (39.4%) |
| Kramer et al. [14] | 2024 | Retrospective | 155 | Masters athletes (≥ 35 years old), 58% were male; mean age: 61 ± 17 years | Athletics, grouped as:  • sprint events (44.5%)  short-distance running (100 m, 200 m, 400 m) and short-distance hurdles  • endurance events (32.9%)  long-distance running and walking (5 km, 10 km, 20 km, marathon, steeplechase, 8 km cross-country running, walking events, 1500 m and 800 m)  • strength and power events (22.6%)  throwing and jumping (shot put, hammer throw, weight throw, javelin, discus, throw pentathlon, long jump, high jump, triple jump and pole vault) | Not performed | 11.78 ± 6.94 hours of mean training load per week and 27.8 ± 18.2 years of mean training years |
| Orlandi et al. [15] | 2022 | Retrospective | 605 | Varied athletes from ages of 7 until 79 years old, 72.2% were male. | Based on COCIS guidelines:  • B: cycling and swimming (33%)  • C: soccer, basketball, and volleyball (49.7%)  • D: athletics (17.2%) | Not performed | Not mentioned |
| Vecchiato et al. [16] | 2024 | Retrospective | 684 | Young athletes (< 18 years old), 64% were male; mean age: 14.87 ± 1.96 years | Based on ESC recommendation:  • mixed sports (70%)  • endurance sports (11%)  • skill sports (4%)  • power sports (15%) | Not performed | 6.27 ± 2.07 hours of mean training load per week |
| Christou et al. [17] | 2024 | Cross-sectional | 54 | 83.3% were male; mean age: 28 ± 17 years | Based on ESC recommendation:  • mixed sports (46.2%)  • endurance sports (31.4%)  • power sports (22.2%) | Not performed | 6.4 ± 3.4 hours of mean training load per week |
| Fyyaz et al. [18] | 2023 | Cross-sectional | 314 | 34.7% were male and 94.6% were Caucasian; mean age: 53.5 ± 8 years | Entire population classified into an endurance sport | No positive late gadolinium enhancement presence were found in any athletes | Not mentioned |
| Ollitrault et al. [19] | 2020 | Prospective | 119 | Athletes aged ≥ 18 years old, 86% were male, and 85% were Caucasian; mean age: 27 ± 12 years | Based on ESC recommendation:  • mixed sports (49%)  • endurance sports (42%)  • skill sports (0.8%)  • power sports (8.4%) | Not performed | 13 ± 4 hours of mean training load per week. The study only included athletes with a training load of ≥ 8 hours per week for the last 6 months |

Table S2. Results extracted from included studies

| Author | Fragmented QRS finding | Further Investigations and Follow-up | Other ECG Manifestations |
| --- | --- | --- | --- |
| Graziano et al. | 831 (29%) in at least one lead excluding aVR with 24,3% occurring in lead V1 and 12% in lead V2.  10 (0.5%) in ≥2 contiguous leads, V2-V4 (n=2), V5-V6 (n=1), I-aVL (n=2), II-III-aVF (n=8).  Based on sex, 8 were male and 2 were female.  Based on intensity, 4, 4, and 2 athletes belongs to the high, moderate, and low intensity group respectively.  Based on the type of sport, 7, 2, and 1 athlete belongs to the mixed, endurance, and skill-based sports respectively.  No statistically significant difference was found regarding age, sex, BMI, training load, and type of sport between athletes with and without fQRS | Further investigations were done on the 10 athletes with fQRS in ≥2 contiguous leads, revealing:   - 1 athlete with fQRS in lead V5, V6, and AVL: - murmur and systolic click - exercise testing reveals no abnormalities - echocardiography reveals a mitral valve prolapse with mild regurgitation - 1 athlete with fQRS in lead II, III, and aVF: - PVBs with RBBB configuration during exercise testing - Further work-up including CMR reveals no abnormalities   Follow-up was conducted in 2023 on 5 out of 10 athletes with with fQRS in ≥2 contiguous leads, where fQRS continues to show, without any occurrence of adverse cardiac events | These were found in the 10 athletes with fQRS in ≥2 contiguous leads:  Group 1   - iRBBB (n=1, 10%) - Early repolarization (n=1, 10%) - LVH (n=2, 20%) - RVH (n=2, 20%)   Group 2   - RAD (n=1, 10%) - cRBBB (n=1, 10%)   Group 3   - Pathological TWI (n=1, 10%)   These were found in the remaining 2130 athletes without fQRS in ≥2 contiguous leads:  Group 1   - iRBBB (n=487, 22.86%) - Early repolarization (n=315, 14.79%) - LVH (n=294, 13.8%) - RVH (n=226, 10.61%) - TWI V1-V3 < 16 yr (n=109, 5.12%) - Sinus bradycardia (n=78, 3.66%) - Junctional rhythm (n=2, 0.09%) - Ectopic atrial rhythm (n=36, 1.69%) - 1^st^ degree AV block (n=17, 0.8%)   Group 2   - LAD (n=2, 0.09%) - LAE (n=7, 0.33%) - RAD (n=5, 0.23%) - RAE (n=22, 1.03%)   Group 3   - Pathological TWI (n=8, 0.38%) - QRS ≥140 ms (LBBB morphology) (n=1, 0.05%) |
| Kramer et al. | 49 (32%) in ≥2 contiguous leads  • anterior leads (V1-V5): 19%  • lateral leads (I-aVL-V6): 2%  • inferior leads (II-III-aVF): 19%  Based on the type of sports, the prevalence was 29% (n=10), 29% (n=15) and 34% (n=24) in strength, endurance, and sprint athletes respectively.  Based on age groups, prevalence was higher in older age groups; 25% (n=32), 39.5% (n=38), 27.9% (n=43), and 34.1% (n=41) in the 35-49 yr, 50-59 yr, 60-69 yr, and >70 yr group respectively.  No significant difference was found between sexes. | Not performed | These were found in the 49 athletes with fQRS in ≥2 contiguous leads:  Group 1   - iRBBB (n=2, 4.08%) - LVH (n=11, 22.45%) - RVH (n=2, 4.08%)   These were found in the remaining 106 athletes without fQRS in ≥2 contiguous leads:  Group 1   - iRBBB (n=3, 2.83%) - LVH (n=11, 10.38%) - RVH (n=5, 4.72%)   Group 2   - cRBBB (n=2, 1.89%)   Group 3   - cLBBB (n=1, 0.94%) |
| Orlandi et al. | N: 47 (7.7%) in ≥2 contiguous leads.  Based on multivariate analysis, older age, males, higher CMI value, and lower E wave were significantly associated with fQRS. This finding most likely were affected by the predominantly male sample base. No significant association were found in multivariate analysis regarding type of sport and BMI with the occurrence of fQRS  Athletes with fQRS have significantly older age, mostly males, significantly lower heart rate, significantly higher values on echocardiography (cardiac mass index, interventricular septum, posterior wall thickness, systolic and diastolic LV volume, aortic root valve, and left atrium diameter) when directly compared to athletes without fQRS. | Not performed | Not mentioned |
| Vecchiato et al. | N: 223 (32.6%)  • 19% only in V1  • 8% in both V1 and V2  • anterior leads (V3-V4): 2%  • septal leads (V1-V2): 34%  • lateral leads (V5-V6-I-aVL): 10%  • inferior leads (II-III-aVF): 7%  Based on multivariate analysis on all athletes, fQRS were significantly associated with age and gender.  Athletes with fQRS are mostly males, and significantly have lower heart rate, wider QRS interval, lower QTc, higher exercise capacity, higher RV function and remodelling when directly compared to athletes without fQRS. Significantly higher LV wall thickness were found in highly trained athletes (training load >8 hours per week) with fQRS compared to highly trained athletes without fQRS. No significant difference between athletes with and without fQRS in regards to type of sport, training load, and blood pressure. | No occurrence of adverse cardiac events during 4.57 ± 2.71 years of follow-up | These were found in the 223 athletes with fQRS in lead V1:  Group 3   - ≥2 PVCs (n=11, 4.93%)   These were found in the 461 athletes without fQRS in lead V1:  Group 3   - ≥2 PVCs (n=21, 4.56%)   No significant difference was found between groups |
| Christou et al. | • N: 34 (63%) in lead III  • N: 27 (50%) in lead V1  • N: 23 (42.6%) in lead aVF  • N: 18 (33.3%) in lead aVL  fQRS in lead V1 is significantly associated with higher training age, lower heart rate, and increased measurement of the proximal portion of RVOT | No occurrence of adverse cardiac events during 2.3 (0.8-4.9) years of follow-up. Increasing number of leads with fQRS were found in athletes, with significant correlation to training age. | Not mentioned |
| Fyyaz et al. | N: 33 (10.5%) in ≥2 contiguous leads  Athletes with fQRS are significantly older, mostly males, with significantly higher LV mass index and RV function compared to athletes without fQRS | Not performed | These were found in the 33 athletes with fQRS in ≥2 contiguous leads:  Group 3   - ≥2 PVCs (n=3, 9.09%)   These were found in the remaining 281 athletes without fQRS in ≥2 contiguous leads:  Group 3   - ≥2 PVCs (n=49, 17.44%)   No significant difference was found between groups |
| Ollitrault et al. | N: 26 (21.8%) in lead V1  Based on multivariate analysis, fQRS are associated with older age and higher RVOT diameter measurement. | Not performed | Not mentioned |
